# Visualization of the omental bursa and its spatial relationships to left subphrenic extraperitoneal spaces by the second Chinese Visible Human model

**DOI:** 10.1007/s00276-015-1462-3

**Published:** 2015-03-28

**Authors:** Haotong Xu, Ke Xiang, Maihong He, Fuzhou Tian, Yi Wu

**Affiliations:** 1 Postdoctoral Workstation, The General Surgery Center of the Peoples’ Liberation Army, Chengdu Military General Hospital, Chengdu, 610083 Sichuan People’s Republic of China; 2 Department of Infection Control, Fuzhou General Hospital of Nanjing Military Command, The PLA, Fuzhou, 350025 Fujian Province People’s Republic of China; 3 Clinical Medical College of Fujian Medical University in Fuzhou General Hospital of Nanjing Military Command, The PLA, Fuzhou, 350025 Fujian Province People’s Republic of China; 4 Institute of Computing Medicine, Third Military Medical University, Chongqing, 400038 People’s Republic of China

**Keywords:** Visualization, Omental bursa, Left subphrenic extraperitoneal spaces, Splenic bare area, Visible Human Project

## Abstract

**Purpose:**

In order to overcome the obstacle that detailed spatial relationships of the omental bursa to its related spaces cannot be displayed clearly by thick-slice sectional anatomical imaging and computed tomography, we designed a new approach to three-dimensional (3D) visualization of the omental bursa.

**Methods:**

By Amira^®^ software, we employed thin-slice cross-sectional images of the upper abdomen retrieved from second Chinese Visible Human datasets to display the spatial relationships of the omental bursa to its related spaces, especially to the left subphrenic extraperitoneal spaces. Moreover, these spatial relationships were presented on 3D sections reconstructed from second Chinese Visible Human images and computed tomography images.

**Results:**

Of importance, the gastric bare area is located among the superior, inferior, and splenic recesses. The appearance of the foramen bursae omenti majoris is the only pathway communicating between the superior and inferior recesses of the omental bursa, and also is the anatomic landmark between the superior and inferior recesses. The splenic recess is surrounded from behind by the splenic bare area and the gastric bare area.

**Conclusion:**

As one of the subphrenic spaces, the omental bursa with its related spaces was imaged three-dimensionally using a visualization technique. Familiarity with the anatomic location and spatial relationships of the omental bursa to its related spaces may be beneficial for the differential diagnosis and intervention, improving outcome.

**Electronic supplementary material:**

The online version of this article (doi:10.1007/s00276-015-1462-3) contains supplementary material, which is available to authorized users.

## Introduction

The omental bursa (OB) is a potential space in the subphrenic area, an irregular space surrounding the caudate lobe of the liver and distributing between the stomach and pancreas on computed tomography (CT) or magnetic resonance images (MRI). Without pathologic fluid collections in the OB in healthy individuals, the OB is normally collapsed. Dehydration and malformation of autopsy specimens exaggerates this collapse. Thus, it is difficult for medical students to appreciate the anatomic morphology of the OB during training. In contrast, thin-slice cross-sections derived from the Chinese Visible Human Project are brightly colored, the images are clear, and histological structures are satisfactorily displayed [15, 16]. Furthermore, the Second Chinese Visible Human dataset (CVH2) sample has a small amount of ascites due to vascular perfusion with formalin. Additionally, the cross-sections from these datasets, other random sections, and three-dimensional (3D) digital models allowed for visualization of complicated anatomic structures, such as ligaments and extravisceral spaces. This 3D visualization technique has also been used to study organs and tissues, as well as in small animal studies [3, 10]. In this study, we used the visualization technique to study the anatomic features of the OB.

A detailed anatomic location of the OB is not displayed perfectly on normal cross-sectional CT scans or multiplanar reconstruction images. However, it is usually seen in patients with ascites, such as in cirrhosis or ovarian carcinoma. Min et al. [11] argued that the retroperitoneal extension of pancreatic fluid in acute pancreatitis could be considered a good demonstration of retroperitoneal anatomy. Similarly, the large volume of peritoneal fluid derived from cirrhosis or ovarian carcinoma may also delineate the anatomy of the OB.

Amira^®^ software has the advantage of demonstrating colorful images of Visible Humans as well as CT images on random sections. This software is an interactive tool for segmentation and visualization of gray-value images and 3D rendering of objects. Therefore, we utilized this software for the current study.

The morphology of the OB, the gastric bare area, and the boundary of each recess of the OB have been explored by gross anatomy and thick-slice sectional anatomical imaging [9, 11, 17]. However, none of these investigations elucidates the exact relationships of the OB to left subphrenic extraperitoneal spaces. According to the radiological scoring system (CT severity index score) developed by Balthazar and colleagues in 1994, the single and ill-defined fluid collection was defined as D grade; two or more poorly defined collections in or adjacent to the pancreas were defined as E grade [2]. Due to the fact that the OB is prone to be involved in acute pancreatitis [1], whether fluid collections in or around the OB have been accurately defined would influence the CT severity index score. In addition, treatment of peripancreatic fluid collections influences mortality and morbidity in acute pancreatitis. Interobserver agreement was found to be poor regarding the Atlanta definitions of the various local complications in 2006 [4]. Using 3D visualization, detailed anatomic division in each OB recess and spatial relationships, especially to the left subphrenic extraperitoneal spaces, can be clearly displayed. This kind of anatomic division will be helpful in determining the correct location of fluid collections in or around the OB in acute pancreatitis. Subsequently, the CT severity index score might be as accurate as possible, helping to optimize management.

OB abnormalities included a variety of pathologic entities: fluid collections due to inflammation, carcinoma, hemorrhage, and traumatic lesions invading the lesser sac [5]. There is a rich lymphatic network around the stomach and pancreas; the lymph nodes within the OB and the omental foramen are usually involved during lymphatic metastasis from gastric or pancreatic carcinoma. Due to nonspecific abdominal symptoms and radiologic characteristics, CT diagnosis of entities involving the OB is difficult [6]. Except when combined with clinical symptoms and CT manifestations involving the OB, familiarity with stereoscopic spatial relationships of the OB to its adjacent structures increases the diagnostic accuracy of OB abnormalities.

We investigated the spatial relationships of each recess of the OB to its adjacent structures, especially to left subphrenic extraperitoneal spaces, in order to provide 3D models of each recess of the OB, the gastric bare area, and the splenic bare area based on the CVH2 datasets. This information may aid in defining the anatomy of the OB for medical education purposes and for the diagnosis of OB abnormalities.

## Materials and methods

The study was approved by the Ethics Review Board of the Chengdu Military General Hospital. The CVH2 datasets were obtained from voluntary donation [15]. Written consent was obtained from relatives of CVH2 participants. CT scans of 53 patients with cirrhosis or ovarian carcinoma were used, although informed consent was waived by the Institutional Review Board of our Hospital; no identifiable information (i.e., age and gender) was used.

### Three-dimensional reconstruction of the OB and left subphrenic extraperitoneal spaces based on CVH2

#### Cross-sectional data of the upper abdomen from the CVH2

CVH2 datasets were from a 22-year-old woman. This cadaver, which was without organic disease, was donated on a voluntary basis. Successive thin-slice cross-sectional images of the upper abdomen with a 170-mm thickness, from the dome of the diaphragm to the lower pole of the left kidney, were retrieved from the CVH2 datasets for 3D reconstruction. Preliminary CT and MRI scans were performed on the cadaver to exclude lesions of the upper abdomen prior to milling. We performed vascular perfusion with a formaldehyde solution until solution extravasated from the nasal mucosa. Simultaneously, a small amount of ascites formed, and some subspaces in the peritoneal cavity became filled with the solution. The cadaver was placed in a supine position, with the median sagittal plane of the upper abdomen parallel to the body’s long axis. The slice interval was 0.5 mm and the resolution was 6,291,456 (3072 × 2048) pixels. Each Tagged Image File Format (TIFF) picture occupied 36 Mb (approximate pixel size 167 μm), as previously described [15].

#### Segmentation

With Photoshop CS^®^ (Adobe Systems Incorporated, USA), images were registered through four reserved fiducial rods; the background was removed. In addition, structures including the superior, inferior, and splenic recesses of the OB, the vestibulum bursae omentalis, foramen bursae omenti majoris, gastric bare area, splenic bare area, upper pole of the left retroperitoneal space, left adrenal gland, stomach, and pancreas were outlined with the magnetic lasso tool. Outlines of different structures were filled with distinct colors using the “fill” command. After changing the 16-bit color to 2-bit color, color-filled images were transferred to gray-scale images. The 340 gray-scale images retrieved from the CVH2, including the above structures without background, were imported to Amira 5.2.0^®^ software (TGS Company, USA) and a new stack file with the expanded name “hx” was created. With the “Thresholding” tool provided by Amira 5.2.0^®^, the above structures were segmented on a threshold basis, and each structure was labeled with a different mask.

#### Superficial outline reconstruction

After segmentation, the 3D Volume Rendering command was used to reconstruct 3D models of each recess of the OB and the left subphrenic extraperitoneal spaces of CVH2. The surfaces of 3D-reconstructed structures were smoothed with the smooth surface command. The models of superficial outline reconstruction of the OB and its related structures were then displayed.

#### Multiplanar reconstruction of the upper abdomen

The above 340 serial cross-sectional images of the upper abdomen from the CVH2 dataset were imported to Amira^®^ to obtain the orthoslices of the upper abdomen via multiplanar reconstruction. The orthoslice images were observed in a successive manner, clearly and directly [15].

### Anatomy of each recess of the OB and spatial relationships to the left subphrenic extraperitoneal spaces

The anatomical relationships of each recess of the OB to their adjacent structures and the spatial relationships of the OB to the left subphrenic extraperitoneal spaces were explored on consecutive cross-sections and the 3D models of the CVH2. In order to observe certain structures clearly and directly, the 3D models of the OB, the gastric bare area, the stomach, and the pancreas were superimposed on consecutive cross-sections or orthoslice sections.

### Omental bursa on CVH2 images versus multislice CT images

Fifty-three patients with cirrhosis or ovarian carcinoma with large peritoneal fluid collections (39 males and 14 females; mean age 37.2 years, range 25–58 years) were recruited to undergo contrast-enhanced 64-slice spiral CT scans (General Healthcare, Milwaukee, WI, USA). Patients met the following inclusion criteria: no history of abdominal disease except for cirrhosis or ovarian carcinoma, and no history of abdominal surgery. Iodine contrast medium was injected (150 ml; 300 mg/ml) through any available superficial vein in the cubital fossa and images were transmitted to Amira^®^ software. Upper abdominal multislice CT coronal or sagittal images were reconstructed using the multiplanar reconstruction technique. The reconstructed thickness of the multiplanar reconstruction was 0.33 mm and the reconstructed interval was 0.33 mm. The spatial relationships among each recess of the OB and their spatial relationships to the gastric bare area, the splenic bare area, the left gastropancreatic fold, the anterior left subhepatic space, the gastrosplenic recess, and the splenorenal recess on multislice CT images were studied and compared with those from the CVH2 images. Both superior and inferior recesses being involved by fluid was defined as the communication between these two recesses. If only the superior recess filled with fluid, it meant that these two recesses were obstructed.

## Results

### Anatomy of the OB

#### Spatial relationships of the superior recess of the OB to its adjacent structures

**Fig. 1:**
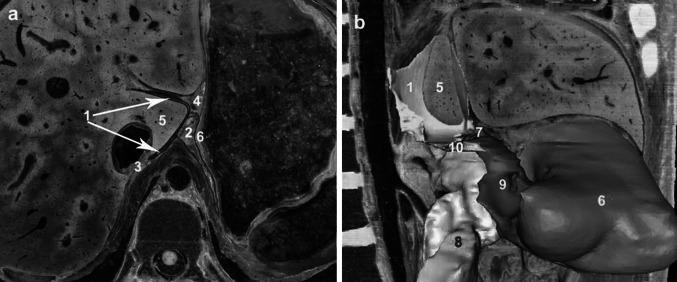
Visualizing the spatial relationships of the superior recess to its related spaces in the CVH2. The superior recess of the OB is imaged cross-sectionally from the inferior aspect (**a**) and in 3D from the right (**b**). *1* Superior recess of the omental bursa, *2* gastric bare area, *3* hepatic bare area, *4* hepatogastric ligament, *5* caudate lobe of the liver, *6* gastric wall, *7* foramen bursae omenti majoris, *8* pancreas, *9* inferior recess of the omental bursa, *10* vestibulum bursae omentalis

On consecutive cross-sections, the superior recess of the OB surrounds the left sides of the caudate lobe of the liver; namely the caudate lobe of the liver is the right border of the superior recess. The right posterior aspect of the superior recess is the left layer of the coronary ligament; in other words, the superior recess is adjacent to the hepatic bare area in this direction. The hepatogastric ligament lies in its anterior direction; the hepatogastric ligament separates the superior recess from the anterior left subhepatic space. The left of the superior recess is contiguous to the gastric bare area (Fig. 1a). The superior margin of the superior recess attaches to the dome of the left diaphragm; the inferior margin is the vestibulum bursae omentalis (Fig. 1b).

#### Communication of the superior and inferior recess of the OB

**Fig. 2:**
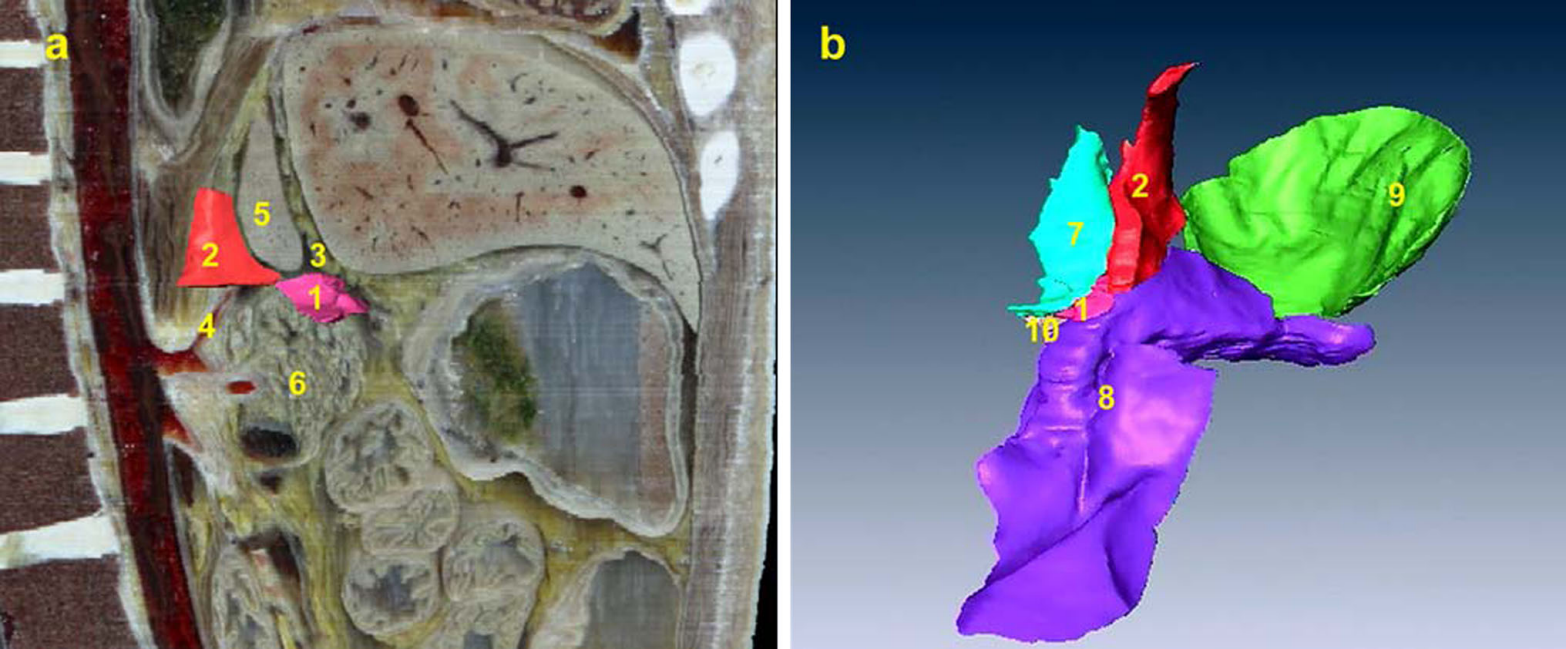
Manifestation of the relationship between the superior and inferior recess. **a** Visualization of the spatial relationships of the foramen bursae omenti majoris to its related spaces from the right. **b** The communication of the superior and inferior recess presented in 3D from the anterior. *1* Foramen bursae omenti majoris, *2* gastric bare area, *3* hepatogastric ligament, *4* left gastric artery, *5* caudate lobe of the liver, *6* pancreas, *7* superior recess, *8* inferior recess, *9* splenic recess, *10* vestibulum bursae omentalis

The left gastropancreatic fold transmits the left gastric vessels from the posterior abdominal wall to the lesser curvature of the stomach. In the case of the CVH2, the anterior margin of the left gastropancreatic fold, the posterior layer of the hepatogastric ligament, and the gastric bare area are not completely adherent to each other; the area located among them is the foramen bursae omenti majoris (Fig. 2a). This foramen is the only pathway to communicate between the superior and inferior recesses of the OB, and is also the anatomic landmark between the superior and inferior recesses of the OB. The upper part of this foramen is the superior recess and the vestibule, and the lower part contains the inferior and splenic recesses (Fig. 2b).

#### Spatial relationships of the inferior recess of the OB to its adjacent structures

**Fig. 3:**
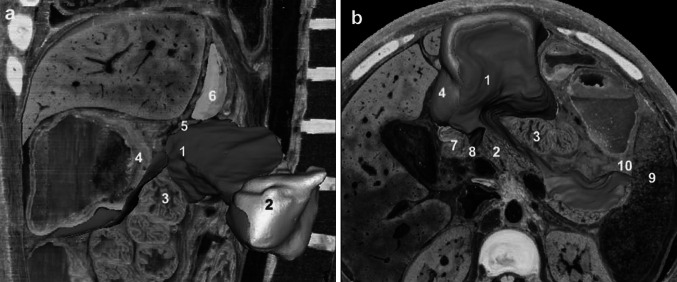
Presentation of the spatial relationships of the inferior recess to its related spaces on the CVH2. The inferior recess is displayed in 3D from the left (**a**) and inferior (**b**). *1* Inferior recess, *2* pancreas, *3* jejunum, *4* gastric wall, *5* foramen bursae omenti majoris, *6* superior recess, *7* beginning of the duodenum, *8* common hepatic artery, *9* spleen, *10* splenic bare area

The inferior recess is located among the posterior wall of the stomach, the transverse colon, the transverse mesocolon, and the pancreas. In the case of the CVH2, the upper boundary of the inferior recess overlays the superior margin of the pancreas. Because the jejunum is located cranially to the stomach and pancreas, the lower boundary of the inferior recess is the jejunum and mesentery. The anterior border is the peritoneum, which covers the posteroinferior aspect of the stomach. The posterior border is the peritoneum, on which lies the anterior part of the pancreas (Fig. 3a). The left border is the splenic recess of the OB, the inner margin of the spleen, and the splenic bare area which is composed of the gastrosplenic ligament, the splenorenal ligament, and the splenocolic ligament. The right border contains the peritoneal reflections below the beginning of the duodenum and the inner margin of the common hepatic artery (Fig. 3b).

#### Spatial relationships of the splenic recess of the OB to its adjacent structures

**Fig. 4:**
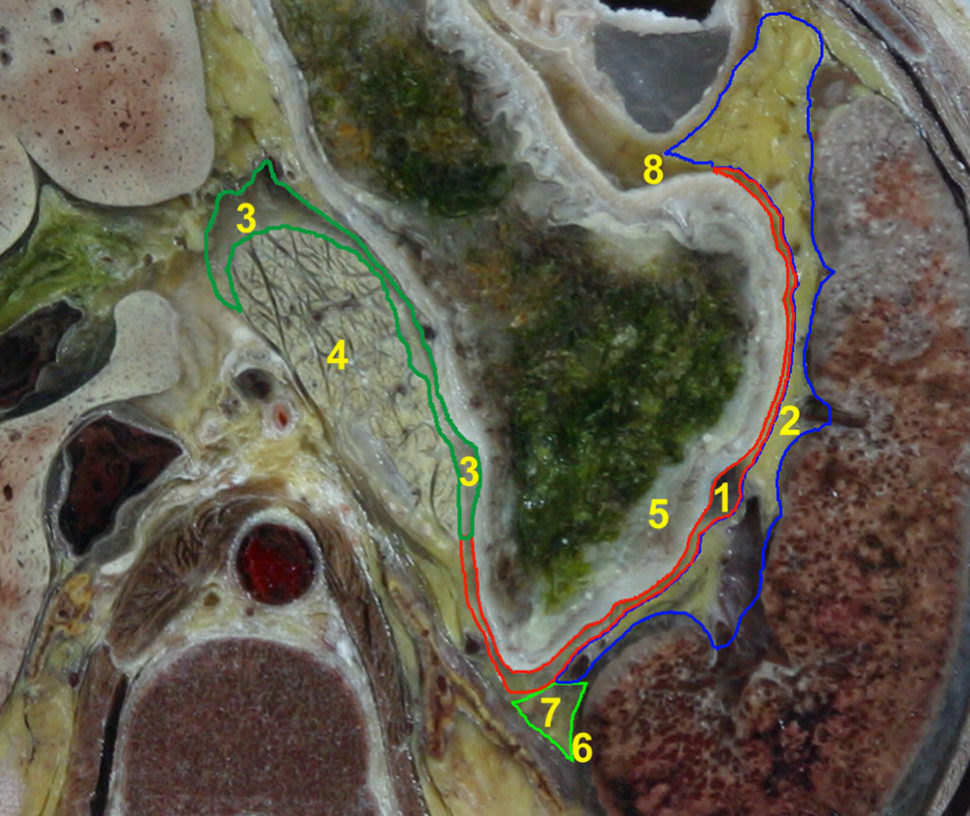
Visualizing the spatial relationships of the splenic recess to its related spaces on the CVH2 from the inferior. *1* Splenic recess, *2* splenic hilum part of splenic bare area, *3* inferior recess, *4* pancreas, *5* gastric wall, *6* splenorenal recess, *7* splenorenal part of splenic bare area, *8* gastrosplenic recess

In the cross-section of the CVH2, the crescentic splenic recess distributes along the greater curvature of the stomach. Medially, the splenic recess surrounds the greater curvature; the splenic bare area serves as the left boundary. The gastrosplenic ligament separates the splenic recess from the gastrosplenic recess; the splenorenal ligament separates the splenic recess from the splenorenal recess. The splenic recess is limited to the anterior boundary by the adhering point of the gastrosplenic ligament on the greater curvature. In the inferomedian direction, the splenic recess is adjacent to the inferior recess (Fig. 4).

### Spatial relationships of the OB to left subphrenic extraperitoneal spaces

#### Spatial relationship between the OB and gastric bare area

In the cross-section, the gastric bare area lies between the superior and splenic recesses of the omental bursa. The gastric bare area is surrounded by the superior, splenic, and inferior recesses of the omental bursa from the anterointernal, posteroexternal, and inferior directions, respectively, in 3D visualization imaging (Fig. 2b).

#### Spatial relationship between the OB and splenic bare area

**Fig. 5:**
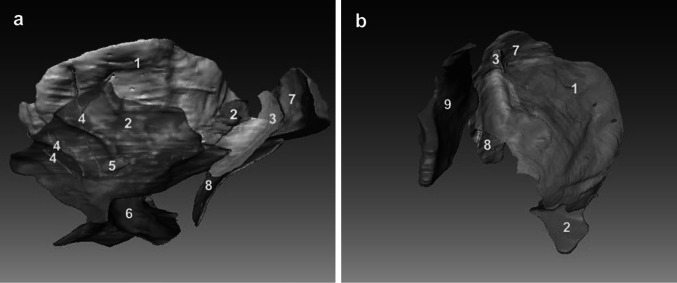
Showing spatial relationships between the OB and the left subphrenic extraperitoneal spaces from lateroinferior (**a**) and anterosuperior (**b**). *1* Splenic recess, *2* splenic hilum part of splenic bare area, *3* splenorenal part of splenic bare area, *4* gastric short veins, *5* splenic hilar lymph node, *6* inferior recess, *7* upper pole of the left retroperitoneal space, *8* left adrenal gland, *9* gastric bare area

The splenic bare area is adjacent to the splenic recess and inferior recess of the OB. This bare area can be divided into the splenic hilum and splenorenal parts. The splenic hilum part is located in the anterosuperior direction of the inferior recess. The splenic hilum part surrounds the vast majority of the splenic recess with a semi-arc shape laterally (Fig. 5a). Meanwhile, the splenorenal part distributes in the posterolateral direction of the splenic recess (Fig. 5b).

#### Spatial relationship between the OB and left retroperitoneal space

The upper pole of left retroperitoneal space lies posteromedial to the splenic recess (Fig. 5b). This upper pole and the splenorenal part of the splenic bare area encircle the latter part of the splenic recess together.

### Omental bursa on CVH2 images corresponding with that on multislice CT images

**Fig. 6:**
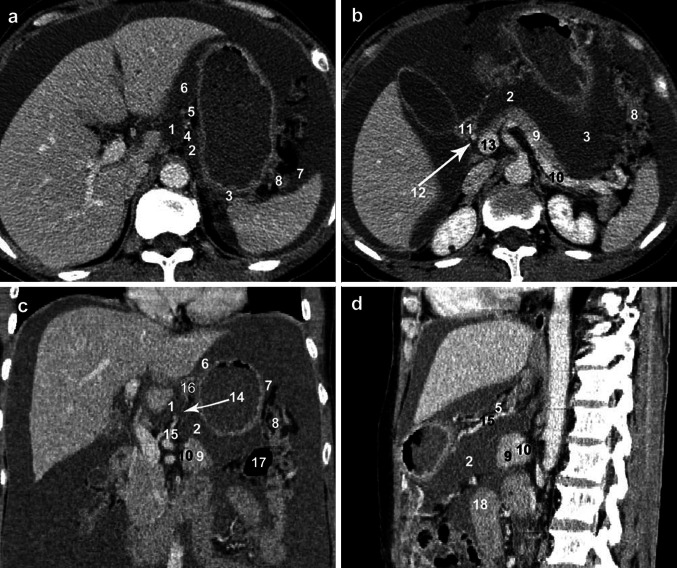
Displaying spatial relationships of OB to its adjoining structures on CT images of a woman with ovarian carcinoma. Visualization of the OB on a higher and lower cross-section (**a**, **b**), coronal (**c**), and sagittal (**d**) images, respectively. *1* Superior recess, *2* inferior recess, *3* splenic recess, *4* left gastropancreatic fold, *5* hepatogastric ligament, *6* anterior left subhepatic space, *7* gastrosplenic recess, *8* splenic bare area, *9* pancreas, *10* splenic artery, *11* beginning of the duodenum, *12* common hepatic artery, *13* portal vein, *14* foramen bursae omenti majoris, *15* left gastric artery, *16* gastric bare area, *17* transverse colon, *18* jejunum

The anatomic location of the OB, its associated vasculature, and its spatial relationships to the adjoining structures on the CVH2 images (Figs. 1a, 4) are well depicted on axial CT images (Fig. 6a, b). The anatomic location of the OB on the CVH2 images is well depicted on coronal and sagittal CT images of patients with ovarian carcinoma created with the multiplanar reconstruction technique (Fig. 6c, d). However, there is no distinct demarcation between the superior recess and the vestibule and there is no distinct boundary between the inferior and splenic recesses; they overlap (Fig. 6a–c) on CT images. The superior and inferior recesses were found to communicate directly with each other in 36 patients (67.9 %, 36/53). The superior recess did not communicate with the inferior recess in 17 patients (32.1 %, 17/53).

## Discussion

Although much research concerning the spatial relationships among subphrenic spaces has been performed during gross anatomy, thick-slice sectional anatomy, and CT imaging [8, 9, 11, 17], thin-slice cross-sections retrieved from the Chinese Visible Human datasets have seldom been used. The cross-sections retrieved from Visible Human datasets provide images of a higher resolution than those obtained from CT or MRI scans, and with more detailed anatomic information compared with those from thick-slice sectional anatomy. This type of cross-sectional imaging, therefore, allows for visualization of the spatial relationships of each recess in the OB to its adjacent spaces.

The combination of visualizing 3D models derived from CVH2 datasets with sectional images of the OB and left subphrenic extraperitoneal spaces will contribute to a better understanding of the spatial relationships of each recess of the OB to their adjacent structures, especially to the left subphrenic extraperitoneal spaces, during anatomy instruction (Figs. 1, 2, 3, 4, 5). Furthermore, 3D models of the OB and left subphrenic extraperitoneal spaces can make up for the disadvantage of two-dimensional images, helping medical students to establish a stereoscopic impression of the OB and the left subphrenic extraperitoneal spaces (Figs. 2b, 5b).

Zhao et al. [17] discovered communication between the superior and inferior recesses can be divided into two types: (1) the superior and inferior recesses communicate directly with each other, as in CVH2; (2) the superior recess cannot communicate with the inferior recess. Our results regarding the percentage of communication between these two recesses were consistent with cadaveric data. Prior research asserted that connective tissue associated the anterior margin of the gastropancreatic fold to the posterior layer of the hepatogastric ligament, and that the connective tissue obstructed these two recesses. In our research, we confirmed that the gastric bare area was surrounded by the superior, splenic, and inferior recesses of the OB from the anterointernal, posterolateral, and inferior directions, respectively, in 3D imaging (Fig. 2b). Except for the connective tissue, we believe that the gastric bare area can obstruct these recesses as well. In some individuals with a large gastric bare area, especially if the position of the tip of the triangular gastric bare area is low enough, the gastric bare area will occupy the space of the foramen bursae omenti majoris; the superior recess will not be able to communicate with the inferior recess [14].

Besselink et al. [4] showed that interobserver agreement in the use of the Atlanta classification to categorize peripancreatic fluid collections on CT was poor, even after assessment by experienced abdominal radiologists. We argue that the main reason was that observers did not come to terms with the anatomic allocation of the same fluid collections most of the time. Even then, the anatomic allocation of peripancreatic fluid collections would influence the CT severity index score and further differentiate between moderate and severe acute pancreatitis. In our study, we confirmed that the superior recess overlaps with the vestibulum bursae omentalis, and that the splenic recess is superimposed on the inferior recess on successive cross-sections of CT images (Fig. 6a, b). Therefore, there is no distinct boundary between the superior recess and the vestibule. Similarly, we cannot distinguish the splenic recess from the inferior recess. We suggest that the space above the foramen bursae omenti majoris that surrounds the caudate lobe should be defined as the medial compartment of the OB, while the space below this foramen distributing between the stomach and pancreas should be regarded as the lateral compartment of the OB on CT images. This kind of division corresponds well with the embryogenesis of the OB [12]. If pancreatic fluid involves only the space below the foramen bursae omenti majoris, we consider it to be one location for fluid collections, and count it into the CT severity index score. When pancreatic fluid involves both the medial and lateral compartments of the OB, we consider them as separate locations. Therefore, clinicians and radiologists should compromise with the anatomic allocation of the OB and its adjacent spaces in clinical practice and research. Only in this way can radiologists assess peripancreatic fluid collections with less difficulty, and CT findings will be more compatible with the pathologic progress of acute pancreatitis. Surgeons and gastroenterologists can rely on the radiologist’s CT report of a patient with acute pancreatitis to determine the optimal management.

The left extraperitoneal space lies anterior to the left suprarenal gland and upper pole of the left kidney in Gray’s Anatomy [13]. On the other hand, the left extraperitoneal space is usually named for the gastric bare area clinically [7]. However, as one of spaces that lie outside the peritoneal coverings in left upper quadrant, the splenic bare area participated in the gastric bare area composing the left subphrenic extraperitoneal spaces (Fig. 5). Namely, the left subphrenic extraperitoneal spaces are composed by the gastric bare area and splenic bare area. The OB has intimate spatial relationships to these areas. Familiarity with the relationships of the splenic recess to the splenic bare area and the gastric bare area will be helpful for locating lesions and fluid collections in the left upper quadrant on CT/MRI, especially in distinguishing lesions of the lateral compartment of the OB from the vasculopathy and lymphadenopathy in the splenic hilum, and large tumors in the upper pole of the left retroperitoneal space.

## Conclusion

In summary, we innovatively used Visible Human Project to present the anatomic location and spatial relationships of the OB to its adjacent structures. The vaguely defined location of the left subphrenic extraperitoneal spaces was clarified by visualization technique. 3D models of the OB may improve the demonstration of the OB anatomy and the detection of the OB pathologic conditions. In practice, the coronal and sagittal reformatted CT/MRI images will help delineate the exact location, origin, or spread pattern of the OB disease, as well as clarify the complex anatomy of the OB.

## Electronic supplementary material

Below is the link to the electronic supplementary material.

Movie S1 200 images are captured for presentation of each recess of the OB with its adjacent gastric bare area, stomach, pancreas, and left adrenal gland that rotate 360° (MPG 4143 kb)

Movie S2 200 images are captured for presentation of a 3D model of each recess of the OB and the gastric bare area that rotate 360° (MPG 3081 kb)

Movie S3 200 images are captured for presentation of inferior and splenic recess with its adjacent gastric bare area, splenic bare area and upper pole of the left retroperitonral space that rotate 360° (MPG 4161 kb)
